# Prevalence of Underweight and Overweight and Its Association with Physical Fitness in Egyptian Schoolchildren

**DOI:** 10.3390/ijerph17010075

**Published:** 2019-12-20

**Authors:** Osama Abdelkarim, Achraf Ammar, Khaled Trabelsi, Hamdi Cthourou, Darko Jekauc, Khadijeh Irandoust, Morteza Taheri, Klaus Bös, Alexander Woll, Nicola Luigi Bragazzi, Anita Hoekelmann

**Affiliations:** 1 Faculty of Physical Education, Assiut University, Assiut 71515, Egypt; osamahalim@ymail.com; 2 Institute of Sport Science, Otto-von-Guericke University Magdeburg, 39104 Magdeburg, Germany; ammar.achraf@ymail.com (A.A.); anita.hoekelmann@ovgu.de (A.H.); 3 High Institute of Sport and Physical Education, University of Sfax, Sfax 3000, Tunisia; trabelsikhaled@gmail.com; 4 Education, Motricité, Sport et Santé (EM2S), High Institute of Sport and Physical Education, University of Sfax, Sfax 3000, Tunisia; 5 Activité Physique, Sport et Santé, UR18JS01, Observatoire National du Sport, Tunis 1003, Tunisia; 6 Institute for Sport and Sports Sciences, Karlsruhe Institute of Technology, 76131 Karlsruhe, Germany; darko.Jekauc@kit.edu (D.J.); klaus.boes@kit.edu (K.B.); Alexander.woll@kit.edu (A.W.); 7 Department of Sport Sciences, Imam Khomeini International University, Qazvin 34148-9681, Iran; irandoust@soc.ikiu.ac.ir (K.I.); taheri_morteza@yahoo.com (M.T.); 8 Department of Mathematics and Statistics, Laboratory for Industrial and Applied Mathematics (LIAM), York University, 4700 Keele Street, Toronto, ON M3J 1P3, Canada; 9 Department of Health Sciences (DISSAL), Postgraduate School of Public Health, University of Genoa, 16132 Genoa, Italy

**Keywords:** BMI categories, physical fitness, schoolchildren, age, gender difference

## Abstract

Underweight and overweight are serious health concerns for many children and could be associated with low physical-fitness levels. This study aimed (i) to evaluate the prevalence of underweight and overweight and (ii) to examine its association with the physical fitness levels in primary male and female schoolchildren. Including 13 government primary-schools, a cross-sectional survey was conducted between 2014 and 2017. Anthropometric characteristics together with the physical-fitness level were measured in 931 schoolchildren aged between 6- and 11-years old. The prevalence of under- and overweight children were 8.49% and 24.06%, respectively. These proportions were not significantly different between males and females and were affected by age (*p* < 0.001), with a higher prevalence of overweight and a lower prevalence of underweight at 9–11 years, compared to 6–8 years old. Concerning the physical fitness levels, statistical analysis showed a better performance among males compared to females, among participants aged 9–11 years, compared to 6–8 years old, and among underweight and normal-weight, compared to overweight children (*p* < 0.001). There was a higher prevalence of overweight and lower prevalence of underweight at 9–11 years compared to 6–8 years old. Physical fitness levels were better in (i) males, compared to females, (ii) schoolchildren aged 9–11 years, compared to 6–8 years old, and (iii) underweight and normal-weight, compared to overweight children.

## 1. Introduction

Childhood overweight and obesity have been considered by the World Health Organization (WHO) as a serious public health challenge, especially in low- and middle-income countries [1]. Egypt is one of the lower middle-income countries (LMICs) in which overweight and obesity among schoolchildren constitutes an emerging concern and has increased from 6% to 15%, between 1990 and 2010 [2,3].

Overweight and obesity in schoolchildren could be related to multiple causes, such as the modern lifestyle characterized by inactivity and passive overeating over past years [3]. Previous studies have suggested that the prevalence of overweight and obesity could differ from birth to adolescence and between males and females [4,5,6,7].

In the United States, Ogden et al. [6] showed that overweight and obese children are likely to stay obese during their adulthood and could develop chronic diseases at a younger age. In Korea, Song et al. [7] showed significant effects of age groups with differences between males and females on overweight and obesity-related quality of life. Similarly, Maruf et al. [5] showed that age significantly affect overweight (i.e., derived from BMI) prevalence in Nigeria and reported that males between the age of 2 and 6 years had a higher BMI than females; while the opposite results were observed in the age group of 11–18 years. Recently, Cruz Estrada et al. [4] showed that among Mexican adolescents aged between 15 and 17 years, females have a higher overweight rate (3:1) and a lower physical fitness performance than males.

Underweight is partly related to under-nutrition (energy expenditure > intake) [8] and is a significant problem in LMICs; however, overweight and obesity are associated with sedentary behavior and over-nutrition (energy intake > expenditure) [9]. Underweight, overweight or obesity is a desirable health status and constitutes the extremes of malnutrition. 

In Egypt, several studies have examined the prevalence of childhood overweight and obesity in some regions in Egypt, such as Cairo [10], Sohaj [2], Assiut [11], Menoufia [3], Sharkia [12], and Port Said [13]. However, none of these previous studies have investigated the prevalence of both overweight and underweight at different periods of childhood or compared them between males and females.

On the other hand, body weight alteration (underweight or overweight) in childhood has been suggested to significantly impact on physical, physiological (i.e., hyperlipidemia, hypertension, abnormal glucose tolerance, and infertility), and psychological health (e.g., depression and anxiety) [14].

However, despite the fact that over a period of several years the physical fitness level has been usually considered as an indicator of the status of physical health [15,16,17], the majority of related health studies, especially in LMICs, have evaluated the effect of increased underweight or overweight prevalence on the physiological parameters (e.g., hypertension, glucose, lipid profile) [18], with a lack of studies investigating the effect of this major health problem on the physical fitness levels [11].

Although age and physical fitness levels [15,16,17,18,19] were shown to affect the children’s BMI status and there is a difference between males and females [4,5], no previous study have sought to examine the possible effect of being underweight or overweight on the physical fitness levels of children, by taking into consideration age and gender. Therefore, given the unsatisfactory data in this field, especially in children from LMICs, the purpose of this study was (i) to examine underweight and overweight prevalence and (ii) to evaluate its effect on the physical fitness level among schoolchildren in Egypt. We hypothesized that age will affect children’s BMI status and their physical fitness levels, with possible differences between males and females.

## 2. Materials and Methods 

### 2.1. Study Design and Sampling

A cross-sectional study was conducted between 2014 and 2017, at 13 government primary schools in the city of Assiut, which is the largest town in upper Egypt and lies about 234 miles south of Cairo. The size of the primary student population with age ranging from 6 to 11 years in Assiut is about 76,334 students. The total number of government schools is 69, distributed over 7 districts. To create an appropriate population representation for the study, a multistage random sampling technique was used to select a sample. At first, 3 government schools from each of the 7 districts of Assiut were randomly selected according to the method of El-said et al. [13], with one school being excluded during the experimental process, owing to some technical problems. Then, based on the different school grade strata, students were selected on the basis of their seating position in the classroom—three desks in the middle, from each row, for all classes in the school [11,12,13]. The final sample was selected randomly from the chosen schools and consisted of 931 children aged 6 to 11 years [between 6 and 8 years: *n* = 354 (males, *n* = 184; and females, *n* = 170); between 9 and 11 years: *n* = 577 (males, *n* = 300; and females, *n* = 277)], in grades 1 to 5 of primary schools in Assiut. 

### 2.2. Data Collection Procedure

After obtaining the ethics approval from Assiut University and the consent of participation from the headmasters and the schoolchildren, data were collected using the following tools.

#### 2.2.1. Anthropometric Measurements 

Evaluation of the anthropometric characteristics of the children was based on three indicators—body height, body weight, and BMI. The instruments were calibrated according to the standard preparation, prior to measurement. Measurements were taken with schoolchildren dressed in light clothing and without shoes, and the follow-up was done by trained research technicians. Weight was measured with a beam balance to the nearest 0.1 kg. Height was measured with a stadiometer to the nearest 0.5 cm. BMI was defined as the ratio of body weight to body height squared, expressed in kg/m^2^. Subjects were classified as underweight, normal weight, and overweight (i.e., overweight and obese) according to the published standards by the International Obesity Task Force, based on age and gender differencecharacteristics [20].

#### 2.2.2. Physical Fitness Test

The German motor test (DMT 6–18) was used to assess the physical fitness of children aged 6 to 18 years [19]. This motor ability test was recommended by the German Association of Sport Sciences (www.sportwissenschaft.de). The different tests were described and performed according to Lämmel et al. [19] procedures. The realization of these tests was achieved through structured motor skills like running, jumping, and balancing. The test was administered in a group setting during regular school classes. Measurements were conducted in different sessions lasting about 90 min. While 5 assistants were participating in the test administration, about 20 children were assessed during each session. The test process was supervised by physical education teachers utilizing the special test equipment. Eight trained testers carried out the tests. After warming up, pupils started with a 20 m sprint, followed by balancing backwards, jumping sideways, push-ups, sit-ups, standing long-jump, and finally a 6-min run. Boys and girls were examined separately, and they were allowed to rest when required. One week before the data-collection test session, a familiarization session with the DMT battery was performed, in order to improve the understanding of the children about their required tasks and to prepare them for the test session.

The content-related validity of the physical fitness was evaluated as being good throughout, with regard to significance and feasibility, as based on expert ratings. Precisely, the test development was based on an international expert questionnaire involving 40 selected fitness experts in 25 European countries who were asked about the relevance of the test contents and requirements in sport-motoric tests, regarding the documentation of Motor Performance Ability (MPA) [19]. To determine test-retest reliability in Egyptian schoolchildren, the motor test was performed twice within 7 days with the same children, applying the same test situation and the same study investigator. There were good test-retest reliability coefficients (*r*-values between 0.68 and 0.94).

### 2.3. Statistical Analysis

All statistical tests were processed using the STATISTICA 13.0 Software (Stat-Soft, Maison-Alfort, France). All values were expressed as mean ± SD. We tested the normality of distributions using the Shapiro–Wilk method before running any statistical test. Two-way analysis of variance (ANOVA) (2 levels [gender—males vs. females] × 2 levels [age—6–8 vs. 9–11 years]) was used to assess the effect of gender and age on BMI values. Three-way ANOVA (2 levels [gender—male vs. female] × 2 levels [age—6–8 vs. 9–11 years] × 3 levels (BMI weight categories—under vs. normal vs. overweight) was used to assess the difference between males and females, age, and BMI weight categories on the physical fitness levels. Least significant difference (LSD) post-hoc test was conducted when significant main effects were found. Effect sizes were calculated as partial eta-squared (η*_p_*^2^) to assess the practical significance of the findings. Significance was set at *p* < 0.05.

## 3. Results

### 3.1. Descriptive Analysis

The present study included 931 school-aged children from Assiut with 484 (52%) were males and 447 (48%) were females (age: 9.1 ± 1.7 years). Anthropometric measurements were as follows: Weight—35.4 ± 11.5 kg for males and 34.8 ± 11.2 kg for females; height:1.37 ± 0.14 m for males and 1.36 ± 0.12 m for females; and BMI:18.4 ± 3.9 kg/m^2^ for males and 18.3 ± 3.7 kg/m^2^ for females. Out of the 931 schoolchildren, 79 (08.5%) were underweight, 628 (67.4%) were normal weight, and 224 (24.1%) were overweight. The prevalence of children who were under-, normal, and overweight among different age groups (6–8 years and 9–11 years) in both males and females is shown in Table 1. Thedistribution was found to be similar in males and females. 

Indeed, the prevalence of overweight children was 23.8% for males (115 out of 484), and 24.4% for females (109 out of 447). The prevalence of underweight children was 8.6% for males (42 out of 484) and 8.3% for females (37 out of 447). The prevalence of normal weight children was 67.6% (327 out of 484) and 67.3% (301 out of 447), respectively, among male and female schoolchildren. According to the age of the studied population, among both males and females, the prevalence of underweight children was highest, at age of 6–8 years (9.8% among males and 12.4% among females), and decreased with the increase in age (8.0% among males and 5.8% among females at 6–8 years); while the number of overweight children increased with age to be highest at 9–11 years, compared to 6–8 years (26% vs. 20.1% in males, and 27.4% vs. 19.4% in females). 

Concerning the difference between males and females and the effect of age on BMI values, statistical analysis showed only a significant main effect of age (F = 78.3, *p* < 0.001, and η*_p_*^2^ = 0.1) with a higher BMI at the age 9–11 years, compared to 6–8 years for both males and females (*p* < 0.001).

### 3.2. Effect of Age, Gender Difference,and BMI on Physical Fitness

**Figure 1:**
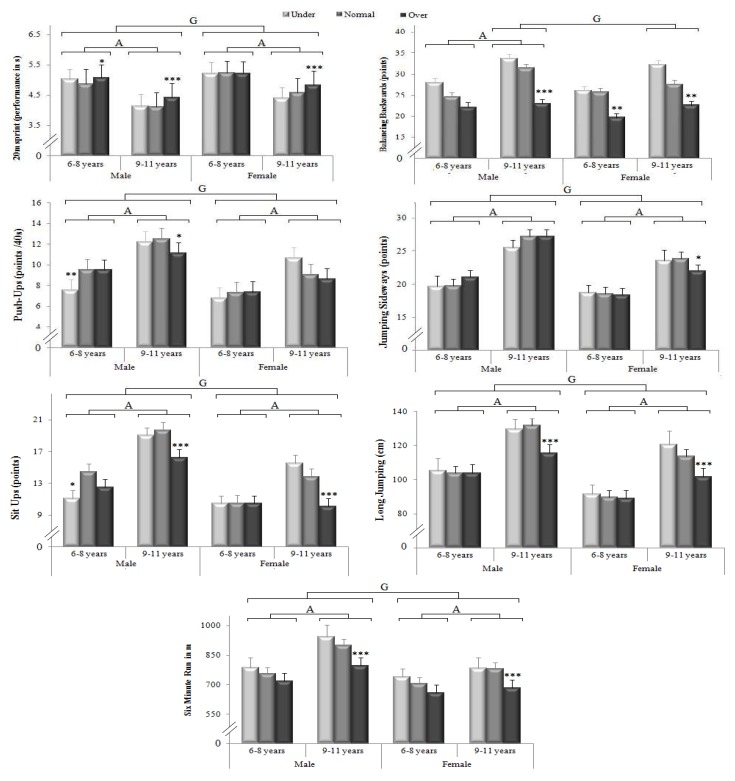
The physical fitness level of the tested population; A—significant difference between 6–8 years and 9–11 years, G—significant difference between males and females; and * significant difference compared to normal weight (*p* < 0.05); ** significant difference compared to normal weight (*p* < 0.01); and *** significant difference compared to normal weight (*p* < 0.001).

The differences between males and females and the effects of age and BMI were examined in several physical fitness tests, including sprint, balance, side jumping, push-ups, long jumping, and 6-min run, as shown in Figure 1. Except the balance test, statistical analysis showed a significant main effect of gender differencein the majority of the physical tests (*p* < 0.001; F = 43.6, and η*_p_*^2^ = 0.05 for sprint, F = 19.4 and η*_p_*^2^ = 0.02 for side jumping, F = 23.5 and η*_p_*^2^ = 0.03 for push-ups, F = 35.6 and η*_p_*^2^ = 0.04 for sit-ups, F = 46.6 and η*_p_*^2^ = 0.05 for long jumping, and F = 42.5 and η*_p_*^2^ = 0.04 for 6-min run). 

Indeed, in both age groups, higher performances were registered among males, compared to female children in all physical fitness test (*p* < 0.001); except balance, in the age group of 6–8 years old, where no-significant (*p* > 0.05) difference between male and female schoolchildren was registered. Regarding the age effect, the data showed that all tested items were affected by age (*p* < 0.001; F = 223.7, and η*_p_*^2^ = 0.2 for sprint, F = 17.1 and η*_p_*^2^ = 0.02 for balance, F = 81.1 and η*_p_*^2^ = 0.08 for side jumping, F = 50.8 and η*_p_*^2^ = 0.05 for push-ups, F = 43.9 and η*_p_*^2^ = 0.05 for sit-ups, F = 111.3 and η*_p_*^2^ = 0.11 for long jumping, and F = 37.9 and η*_p_*^2^ = 0.04 for 6-min run), with higher performances in the age group of 9–11 years, compared to 6–8 years old. This significant main effect of age was shown in both male and femaleparticipants, in all tested abilities (*p* < 0.001); except balance among the female group, where no-significant difference was shown between 6–8 years and 9–11 years (*p* > 0.05).

With respect to the effect of BMI, the result showed a significant main effect of BMI on sprint (*p* < 0.001; F = 10.7; η*_p_*^2^ = 0.02), balance (*p* < 0.001; F = 24.9; η*_p_*^2^ = 0.05), sit-ups (*p* < 0.001; F = 8.7; η*_p_*^2^ = 0.02), long jumping (*p* < 0.001; F = 8.9; η*_p_*^2^ = 0.02), and 6-min run (*p* < 0.001; F = 19.5; η*_p_*^2^ = 0.04), with higher performances in the under and normal weights, compared to the overweight. However, no-significant BMI effect was shown for side jumping and push-ups (*p* > 0.05). These results suggest that balance is affected more by age and BMI, side jumping and push-ups are affected more by age and gender, while the rest of tested items (i.e., sprint, sit-ups, long jumping and 6-min run) are affected in the same way by age, gender, and the BMI weight categories.

More precisely, the results showed that the effect of BMI weight categories on physical fitness tests appeared to be more observable in the age group of 9–11 years, compared to the 6–8-years old. Indeed, among both male and female populations, normal weight showed a higher performance than overweight in long jumping, sit-ups, and 6-min run (*p* < 0.001), only in the age group of 9–11 years. Similarly, higher performances for normal weight compared to overweight children were registered in balance (*p* < 0.001) and push-ups (*p* < 0.05) among males, and in sprint (*p* < 0.001) and side jumping (*p* < 0.05) among females, only in the 9–11 years age group. It should be noted that in all tested abilities, highest performances were registered in the age group of 9–11 years, among underweight and normal-weight males, while lowest performances were registered in the age group of 6–8 years among overweight females.

## 4. Discussion

The aims of the present study were (i) to evaluate the prevalence of underweight, normal-weight, and overweight conditions among primary school children in Assiut, with respect to the differences between males and females and specific age groups, and (ii) to assess the influence of age, gender, and BMI on physical fitness. Results showed an alarming rate ofunderweight (8.5%) and overweight (24.1%) among the Egyptian schoolchildren. Among both males and females, BMI and overweight prevalence increased with age, while underweight prevalence followed an inverse curve. Regarding the physical fitness levels, for the majority of tested items, the results showed—(i) higher fitness for males in both age groups compared to females, (ii) higher fitness in the age group of 9–11 years compared to 6–8years for both males and females, and (iii) lower fitness levels for overweight compared to underweight and normal-weight children, in both male and female groups.

Current studies in the Eastern Mediterranean Region (EMR) showed that the prevalence of overweight among school-aged children (6–18 years old) reached an alarming level [21], with highest percentage registered in Kuwait (32%) [22]. The results of the present study confirm this alarming rate among Egyptian children from Assiut and showed that the overall prevalence of being overweight (i.e., including obese subjects) among children from 6–11 years was 24.06%.

The results are in line with previous studies conducted among different city from Egypt such as in Cairo (i.e., 10.3% overweight + 12.3% obese) [10], Port Said (i.e., 17.7% overweight + 13.5% obese) [13], Sharkia (i.e., 20% overweight + 10.7% obese) [12], and Sohag (i.e., 16.5% overweight + 14.6% obese) [2]. Additionally, according to previous UNICEF reports [23], the prevalence of being overweight among children is increasing in both developed and developing countries; but with different speed patterns. Concerning the EMR, it was estimated that the proportion of overweight school children has almost doubled each decade [21]. 

The comparison between the present overweight proportion among Egyptian children in 2013–2015 (≈ 24%) and the one published by the National Nutrition Institute among Egyptian children in 2004 (≈ 13%) confirmed the the rapid increase in the prevalence of overweight in EMR (e.g., Egypt). This rapid increase in the prevalence of overweight during the last decades in EMR have been generally related to the adaption of modern lifestyles characterized especially by increasing energy intake and inactivity classified as convincing evident factors that might promote overweight [24].

Indeed, the economic improvement over the last 30 years in most EMR countries has resulted in nutrition transitions/change in food consumption, based on energy-dense diets that are higher in fats (i.e., saturated fat, cholesterol, and refined carbohydrates), and are low in polyunsaturated fatty acids and dietary fiber [21,22,23,24]. This nutrition trend accompanied with a sedentary lifestyle and increased levels of stress have consequently resulted in a steep rise in weight [21]. 

Otherwise, the present results showed that the prevalence of underweight among school-aged Egyptian children from Assiut was 8.49%. This proportion seemed to be higher, compared to developing countries [15] and might confirm the under-nutrition (energy expenditure > intake) problem in Egypt [23,24,25]. Indeed, high rates of underweight (23.5%), stunting (16.6%), wasting (17%), malnutrition manifestations (30%; 16.1% as present + 13.9% as suspicious manifestations), and anemia (18.2%; 10.1% as mild anemia (10–10.9 gm) + 10.1% as moderate anemia (7–9.9 gm)), especially in extended families from rural areas has been reported [25]. Also, high prevalence of stunting (22%) and wasting (10%) among Egyptian children were reported by the UNICEF [23].

Also, the present results showed a significant effect of age with increased prevalence of overweight and decreased prevalence of underweight in 9–11-years old, compared to 6–8-years old, for both the male and female groups. Previous studies in the EMR that investigated the prevalence of overweight among various age groups, showed an increased proportion of overweight subjects with increasing age [21]. These proportions ranged from 1.9% to 21.9% among preschool children (<5 years), and increased among schoolchildren to range from 7% to 45%, and doubled (in almost all studies) during adult age, ranging from 25% to 81.9% [20]. Although some data of EMR studies includes a wide age range in school-aged children (6–18-years old), without taking into consideration the pre-and puberty stage, which might differently influence weight gain, the findings of these studies with the present results support previous suggestions that obese children of school pre-puberty age have a high chance of remaining obese in adulthood and of developing associated risks of chronic health problems [7,26]. Thus, childhood could be considered as a key stage of life in which intervention against weight gain is necessary to promote both health benefits [27].

The results of the present study showed that the prevalence of overweight was not different between males and females in all age groups. The difference between males and females on the prevalence of childhood overweight is still under debate [5], especially in EMR. Indeed, studies conducted in Bahrain [28], Tunisia [29], Kuwait [22], and Qatar [30], found that the prevalence of overweight was higher among girls compared to boys. However, boys showed higher overweight prevalence in countries such as Lebanon [31] and United Arab Emirates [32]. 

The present results confirmed the above-mentioned theory, and showed that among Egyptian school-aged children, the prevalence of overweight shows similar trends in males and females in both age groups of 6–8 years (20.1% and 19.4%, respectively) and 9–11 years (26% and 27.4%, respectively). Divergence between the findings could be explained by differences in geographical locations of the populations of these studies, with distinct genetic, environmental, and socio-cultural backgrounds [31] that could probably contribute to the adopted lifestyle (healthy vs. unhealthy). 

It is widely accepted that low physical fitness levels among kids and teenagers also represents a public health issue. Booth et al. [33] showed that physically active children with higher physical fitness levels are more able to maintain a normal weight with enhanced cardiovascular and metabolic health; while children who have lower physical fitness levels are more physically inactive and more likely to be exposed to overweight and cardiovascular health problems. These findings suggest that being overweight could result in lower physical fitness levels and vice-versa. The results of the present study confirmed this link among Egyptian children and showed a significant main effect of BMI on the majority of physical fitness tests in both males and females, with a lower performance for overweight compared to underweight and normal-weight children. However, this effect appears to be more observable in the tested physical fitness items in the age group of 9–11-years (affecting 7 physical fitness tests) compared to 6–8-years old (affecting only 4 test items).

Previous studies have showed that childhood physical fitness levels are significantly related to BMI values [17,34], more precisely it was found that children with higher BMI values have lower levels of physical fitness [4,11,15]. Based on these findings, the negative impact of overweight on the physical fitness levels widely observed in the age group of 9–11-years compared to 6–8-years old could be explained by the difference in BMI values between these age groups.

Indeed, the mean BMI values registered in all weight categories and the specific BMI values registered among the overweight subjects were shown to be significantly increased from 6–8 years to 9–11 years of age, with rates ≈ 12%—16.9 ± 3.2% vs. 19.2 ± 3.9% and 21.6 ± 2.8% vs. 24.4 ± 3.1%, respectively. The present results suggest that even among overweight children, decreasing BMI values could help in enhancing the overall physical fitness levels by decreasing the number of physical fitness items that were shown to be negatively affected by being overweight (e.g., 6-min run, standing long-jump, and jumping sideways).

Concerning the age and the difference between males and females on the physical fitness levels, in the majority of the tested items (except balancing backward), the present results showed a higher performance among males, compared to females in both age groups, and a higher performance in the age group of 9–11 years compared to 6–8 years, in both males and females. The higher overall performance registered in the age group of 9–11-years old compared to 6–8-years old, support the conclusions found in previous research work among German [19] and American children [35], which noted that the physical fitness level is developed and improved with increasing age, over the years of primary school study (6–12-years old). 

The present significant difference between males and females on the level of physical fitness among Egyptian children is similar to that of other studies on children and teenagers from Mexico [4] and Germany [15], which showed that males had a better overall physical fitness performance compared to females, especially in items that aredependent on energy, such as strength (e.g., handgrip and jumping tests), sprint (30 m sprint), and endurance tests (6 min). Taken together, these findings suggest that gender differences (i.e., difference between males and females) and age variables are more likely to affect the performance of physical fitness tests, which is moredependent on energy than on coordination (based on information-oriented abilities). Muscular performance was previously shown to be inversely associated with cardiovascular mortality and ischemic coronary events [36,37,38].

Indeed, a decrease in muscular strength and endurance capacity was accompanied by overweight and obesity [37], dyslipidemia [38], arterial stiffness [36], and less cardio-respiratory capacity [38]. The present differences in the overall physical fitness between Egyptian male and female children suggest that males possess higher cardio-respiratory capacity while females could be affected more easily by cardiovascular diseases. The exact underlying mechanism of this difference is still not well-understood, however, the sexual dysmorphism characterized by higher BMI and percentages of fat values among females on all age groups could be one of the causes [4].

One possible limitation for the present study was that the time of day of measurement was not taken into account. As previous studies have reported that physical and cognitive performance of children is dependent on time-of-day [39,40,41,42,43], further studies are needed to assess the physical fitness at the same daytime and during the same season.

## 5. Conclusions

The present study showed that underweight and overweight are serious problems for Egyptian schoolchildren, particularly in the 9–11-years old age group. These abnormal weight categories are showed to be associated with a low level of physical fitness. Therefore, physical education teachers, parents, scientists, and coaches should be aware of the higher prevalence of underweight and overweight and its effects on physical fitness levels. Thus, a national multi-domains intervention strategy against abnormal weight is needed to promote physical wellness among Egyptian children.

## Figures and Tables

**Table 1:** Characteristics and prevalence of underweight, normal-weight, and overweight in both male and female participants.

Gender	Age	Underweight		Normal-Weight		Overweight		Height	Weight	BMI
	(years)	*N*	%	*N*	%	*N*	%	(m)	(Kg)	(Kg/m^2^)
**Males**	Total = 484 (52%)	42	8.60%	327	67.60%	115	23.80%	1.37 ± 0.14	35.38 ± 11.5	18.42 ± 3.93
	6–8 = 184 (38%)	18	9.80%	129	70.10%	37	20.10%	1.28 ± 0.08	28.53 ± 7.61	17.17 ± 3.25
	9–11 = 300 (62%)	24	8%	198	66%	78	26%	1.43 ± 0.09	39.57 ± 11.3	19.18 ± 4.11 *
**Females**	Total = 447 (48%)	37	8.30%	301	67.30%	109	24.4%	1.36 ± 0.12	34.77 ± 11.24	18.26 ± 3.77
	6–8 = 170 (38%)	21	12.40%	116	68.20%	33	19.40%	1.26 ± 0.07	26.90 ± 7.37	16.75 ± 3.22
	9–11 = 277 (62%)	16	5.80%	185	66.80%	76	27.40%	1.42 ± 0.09	39.53 ± 10.58	19.17 ± 3.78 *
**Total**	6–11 = 931(100%)	79	8.49%	628	67.45%	224	24.06%	1.36 ± 0.12	35.07 ± 11.37	18.34 ± 3.86

* Significant difference compared to 6–8 years.
